# A novel splice site indel alteration in the *EIF2AK3* gene is responsible for the first cases of Wolcott-Rallison syndrome in Hungary

**DOI:** 10.1186/s12881-020-0985-6

**Published:** 2020-03-27

**Authors:** Andrea Sümegi, Zoltán Hendrik, Tamás Gáll, Enikő Felszeghy, Katalin Szakszon, Péter Antal-Szalmás, Lívia Beke, Ágnes Papp, Gábor Méhes, József Balla, György Balla

**Affiliations:** 1 HAS-UD Vascular Biology and Myocardial Pathophysiology Research Group, Hungarian Academy of Sciences, 98, Nagyerdei krt, Debrecen, H-4032 Hungary; 2 Department of Pathology, Faculty of Medicine, University of Debrecen, 98, Nagyerdei krt, Debrecen, H-4032 Hungary; 3 Department of Pediatrics, Faculty of Medicine, University of Debrecen, 98, Nagyerdei krt, Debrecen, H-4032 Hungary; 4 Department of Laboratory Medicine, Faculty of Medicine, University of Debrecen, 98, Nagyerdei krt, Debrecen, H-4032 Hungary; 5 Division of Nephrology, Department of Internal Medicine, Faculty of Medicine, University of Debrecen, 98, Nagyerdei krt, Debrecen, H-4032 Hungary

**Keywords:** Wolcott-Rallison syndrome, *EIF2AK3* gene, Endoplasmic reticulum stress, PERK protein, Splice site variant, Indel alteration

## Abstract

**Background:**

Wolcott-Rallison Syndrome (WRS) is a rare autosomal recessive disease that is the most common cause of neonatal diabetes in consanguineous families. WRS is caused by various genetic alterations of the Eukaryotic Translation Initiation Factor 2-Alpha Kinase 3 (*EIF2AK3*) gene.

**Methods:**

Genetic analysis of a consanguineous family where two children were diagnosed with WRS was performed by Sanger sequencing. The altered protein was investigated by in vitro cloning, expression and immunohistochemistry.

**Results:**

The first cases in Hungary, − two patients in one family, where the parents were fourth-degree cousins - showed the typical clinical features of WRS: early onset diabetes mellitus with hyperglycemia, growth retardation, infection-induced multiple organ failure. The genetic background of the disease was a novel alteration in the *EIF2AK3* gene involving the splice site of exon 11– intron 11–12 boundary: g.53051_53062delinsTG. According to cDNA sequencing this created a new splice site and resulted in a frameshift and the development of an early termination codon at amino acid position 633 (p.Pro627AspfsTer7). Based on in vitro cloning and expression studies, the truncated protein was functionally inactive. Immunohistochemistry revealed that the intact protein was absent in the islets of pancreas, furthermore insulin expressing cells were also dramatically diminished. Elevated GRP78 and reduced CHOP protein expression were observed in the liver.

**Conclusions:**

The novel genetic alteration causing the absence of the EIF2AK3 protein resulted in insufficient handling of severe endoplasmic reticulum stress, leading to liver failure and demise of the patients.

## Background

Wolcott-Rallison syndrome (WRS, OMIM 226980) is a rare autosomal recessive disorder, first described in 1972 [1]. The leading clinical symptom of the disease is persistent neonatal diabetes mellitus that requires insulin treatment. Diabetes develops typically not later than 6 months after birth, but delayed cases have been also described [1–5]. The poor prognosis of WRS is associated with liver failure triggered by endoplasmic reticulum stressors like viral-, bacterial infections, and hypoglycemia; later the outcome is determined by the possible complications of insulin therapy and hepatic injury. The cause of death is multiorgan failure, liver and kidney insufficiency. The broad diversity of the clinical manifestation of WRS consists of spondylo-epiphyseal dysplasia resulting in growth retardation and short stature [1–6]. Developmental delay, osteoporosis, anemia and neutropenia are rare manifestations [1–6]. Only sporadic cases were described with hypothyreoidism [7], exocrine pancreas insufficiency [8, 9], skin [1] or central nervous system abnormalities (microcephaly, pachygyria, mental retardation) [10–13].

The genetic base of the disorder is well-characterized; variants in the Eukaryotic Translation Initiation Factor 2-Alpha Kinase 3 (EIF2AK3) - or traditionally PKR-like endoplasmic reticulum kinase (PERK) - are present in all of the cases [14]. The different forms of the altered gene may present different levels of gene expressions with variable extent of the protein function. Upon endoplasmic reticulum (ER) stress EIF2AK3 detects the accumulation of misfolded proteins in the ER, phosphorylates EIF2α and downregulates the rate of protein synthesis [15]. EIF2α phosphorylation results in the expression of CHOP, a multifaceted transcription factor of ER stress. If the gene is altered and the EIF2AK3 protein is absent or non-functional, large amount of misfolded proteins are accumulating in affected cells and tissues. In cases of WRS the alteration leads to proinsulin aggregation and β cells apoptosis in pancreas. Other organs, mainly liver and kidney also demonstrate the signs of ER stress [16, 17].

Our two presented cases - the first WRS patients in Hungary - had different longevity, although they were siblings living in the same environment and treated by the same medical team. Their parents are fourth-degree cousins of a family in Hungary with Romani (alias Roma) ethnicity. The disease was caused by a novel splice site variant resulting in a non-functional truncated protein, characterized at the DNA, RNA, and protein level. The cause of their death was liver damage leading to multiorgan failure at 4.2 year and 4 month of age. We were able to document extreme endoplasmic reticulum stress even in the younger child. Our aim was to provide data on ER stress in WRS in order to address the hypothesis that ER stress inhibitors could prove beneficial for patients until the en bloc liver-pancreas transplantation.

## Methods

### Patients and controls

**Fig. 1:**
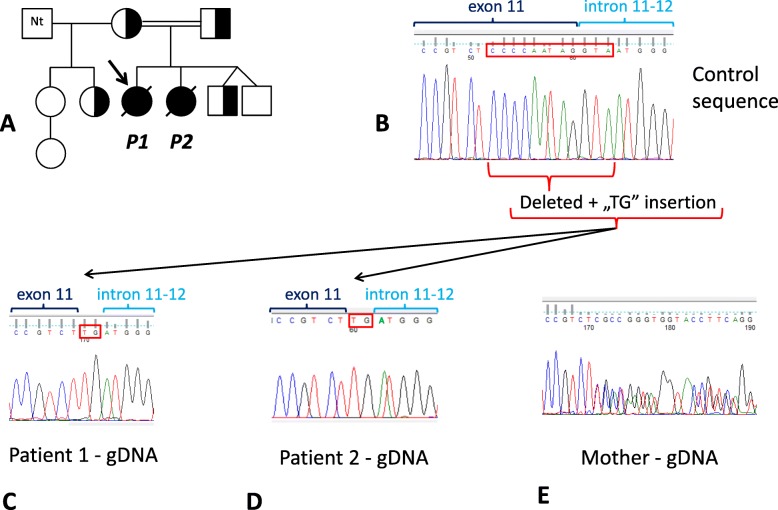
Genetic alteration in the members of the affected family determined by DNA analysis. Altogether 9 of the presented 10 family members were subject of the genetic analysis (**a**). Compared to the wild type sequence (**b**) *Patient 1* was homozygous (**c**) while the mother (**e**), father, one of the brothers and one of the half-sisters were heterozygous carriers of the observed genetic alteration [g.53051_53062delinsTG (Ref Seq: NG_016424.1)] based on genomic DNA (“gDNA”) sequencing using DNA isolated from peripheral leukocytes. In the case of *Patient 2*, only formalin fixed paraffin-embedded tissues were available, therefore, DNA was isolated from these archived liver sections and the region of the variation was amplified by a special PCR. Sequencing of these amplified fragments proved that *Patient 2* was also homozygous for the genetic alteration present in the family (**d**). The red boxes show the deleted nucleotides (**b**) and the inserted “TG” base pair (**c**,**d**). Nt: Not tested

The index patient (*Patient 1*) and her sister (*Patient 2*) showed the typical clinical signs and symptoms of Wolcott-Rallison syndrome and expired due to multiple organ failure induced by viral infection at 4.2 years and 4 months of age, respectively. Their parents were fourth-degree cousins. The parents also have two younger twin sons without any signs of WRS. Additionally, the mother has two older daughters and one grand-daughter - from her former relationship – who are all healthy. Altogether 9 of the described 10 family members were subjected to genetic analyses (Fig. 1a). The control samples used for immunohistochemistry were the routine histological samples of a 43-year-old female person, who died because of an acute fulminant incarceration of truncus cerebri caused by an aneurysm rupture of the arteria communicans anterior. She had no other diseases based on the autopsy results.

### DNA and RNA isolation

Genomic DNA and RNA were isolated from peripheral blood of the still living *Patient 1*, living parents, children and a grandchild, using the QIAmp DNA and RNA Blood Mini kits (Qiagen, Valencia, CA). Since the sister of the index child died years before the diagnosis, formalin-fixed, paraffin-embedded liver tissues were used as source of DNA in her case and the “Tissue protocol” of the QIAmp DNA mini kit (Qiagen) was applied to extract DNA.

### Sequence analysis of the *EIF2AK3* gene from DNA and RNA samples

The coding regions and the exon-intron boundaries of 17 exons of the *EIF2AK3* gene were amplified in 18 PCRs. We designed the PCR primers by Primer 3 software [18] with the exception of exon 3, where we used a primer pair reported in a previous publication [17].

RNA isolated from white blood cells of *Patient 1* and her mother was reverse transcribed using the High-Capacity cDNA Reverse Transcription Kit (Thermo Fisher Scientific, Waltham, MA USA). We amplified the region surrounding the observed genetic alteration from cDNA using a special primer pair designed including sequences of exon 11 and exon 12. The PCR product was 201 bp.

Since DNA isolated from formalin fixed paraffin-embedded tissue contains smaller, fragmented DNA, special PCR reaction was designed surrounding the involved exon-intron boundary of exon 11. The PCR provided a smaller product (129 bp).

The PCR products were sequenced using a cyclic chain-termination sequencing reaction with the BigDye Terminator kit (v.3.1 and v.1.1; Thermo Fisher Scientific) and the ABI PRISM 310 Genetic Analyzer (Thermo Fisher Scientific), as described before [19].

All of the mentioned primer sequences and the PCR conditions are available upon request.

### EIF2AK3 kinase domain expression

The cDNAs of the kinase domains (amino acid 536–1116) of wild type and truncated EIF2AK3 were amplified using the Phusion Hot Start II Polymerase (Thermo Fisher Scientific). Amplimers were ligated into pTriex4Neo expression vector (Merck-Millipore) including an N-terminal 6 × His tag and were transformed into *E. coli* Rosetta2 pLysS (Merck-Millipore). Bacteria were cultured in LB Broth with 100 μg/mL ampicillin, induced with 1 mM IPTG. Cells from an uninduced culture after 5 h of culturing as well 2 and 5 h after the induction with IPTG were taken and centrifuged.

### Western-blot analysis of the EIF2AK3 kinase domain

Pellets of bacteria were lysed in cold lysis buffer and centrifuged. Protein samples were subjected to SDS-PAGE using 12% Tris-glycine gels and were transferred to PVDF membranes (BioRad Hungary, Budapest, Hungary). Anti-His monoclonal antibody (Proteintech, Manchester, United Kingdom), a peroxidase-conjugated goat anti-mouse IgG secondary antibody (GE Healthcare, Waukesha, WI, USA) and the SuperSignal West Pico Chemiluminescent Substrate (Thermo Fisher Scientific) were used for identification of the expressed proteins.

### Immunohistochemical staining

Both the patient and the control tissue samples were fixed with PBS buffered formaldehyde solution and embedded in paraffin wax. The slides were then deparaffinated using xylol and ethanol. Endogenous peroxidase was inactivated (EnVision™ FLEX Peroxidase-Blocking Reagent; Dako, Glostrup, Denmark) and heat-induced epitope retrieval was performed in antigen retrieval buffer solution (RE-7119, Leica, Wetzlar, Germany) in a pressure cooker. Samples were incubated with anti-insulin (clone: rabbit polyclonal 5267650001 Roche Mo. Kft); with anti-PERK (clone: rabbit polyclonal ab79483 - Abcam); with anti-GRP78/BIP (clone: rabbit polyclonal 11587–1-AP - Proteintech Europe); and with anti-CHOP (clone: rabbit polyclonal 15204–1-AP - Proteintech Europe) primary antibodies. The specimens were then incubated with EnVision™ Flex+ Rabbit linker, and EnVision™ Flex/HRP enzyme. Detection of antibody binding was done by incubation with the EnVision™ FLEX/HRP (Dako). Slides were then reacted with DAB (EnVision™ FLEX DAB+ Chromogen - for insulin and PERK) or with VIP (UltraView Universal Alkaline Phosphatase Red Detection Kit 760/503–505 - for CHOP and GRP78) solution. The intensity and distribution of the staining were assessed by light microscopy (Leica DM2500 microscope, DFC 420 camera and Leica Application Suite V3 software, Leica). The slides were counterstained with Gill Hematoxylin solution (105175 Merck Millipore, Billerica, Massachusetts, USA).

## Results

### Clinical cases

#### Patient 1

*Patient 1* was a term newborn, with 2700 g birth weight and 47 cm length. Her mother and father were 28 and 47 years old, respectively, at her birth. This girl was the first child of this relationship, but the mother had two healthy daughters from her previous partner (Fig. 1a). The parents were 4th degree cousins.

The first symptom oral candidiasis, presented at birth, and severe dermatitis was noted at 2 weeks of age. Medical assistance was sought only at 4 months of age. Laboratory tests proved diabetes mellitus with hyperglycemia (23.6 mmol/L), elevated HbA1c (13.7%) and fructosamine (551 μmol/L). Severe leukopenia (3.52 G/L) and neutropenia (0.33 G/L) were also measured. Tests for glutamate decarboxylase and pancreatic islet cell antibodies were negative. Thyroid function was mildly affected, documented with normal sTSH and fT3 but reduced fT4 (10.7 pmol/L).

**Fig. 2:**
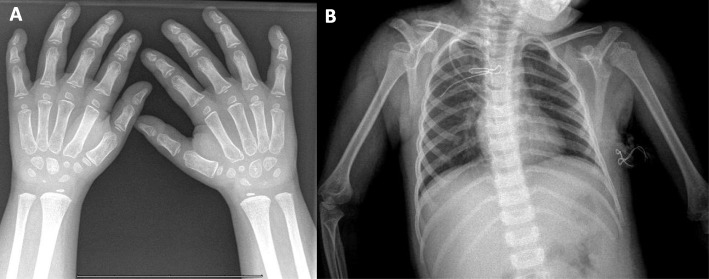
Characteristic signs of spondylo-epiphyseal dysplasia on chest-humeri and arm X-rays. Hand (**a**) and Chest-humeri (**b**) X-rays of *Patient 1* are presented. Short and broad metacarpi and phalanges, porotic bones, hypoplastic bony islands of the basal phalanges below the epiphyseal plates are clearly visible. The epiphyses of the humeri are broadened, the vertebrae have normal morphology

Growth retardation was noticed at 3.2 years of age, with a weight of 12.8 kgs (10–25 percentile) and height of 88 cms (3 percentile). X-rays evaluation revealed signs of spondylo-epiphyseal dysplasia: the epiphyses of the long bones were wide, the phalangeal and metacarpal bones were irregular, short, stubby and porotic, the bony islands below the epiphyseal plates appeared as narrow dots (Fig. 2). The vertebrae showed normal morphology. Psychomotor development was retarted.

The child suffered multiple infectious episodes. Sepsis and liver failure developed at 10 months of age with leukopenia (5.29 G/L) and neutropenia (0.02 G/L). Autoimmune hepatitis was ruled out. At 3.9 years of age, another severe episode of liver failure developed with neutropenia, anemia and renal insufficiency triggered by herpes simplex virus 1. The patient died at 4.2 years of age as a consequence of rapidly progressive multiorgan – primarily liver – failure.

#### Patient 2

*Patient 2* was the younger daughter of the same couple; she was born as a growth restricted newborn with 1920 g birth weight and 43 cm length at 42 weeks of gestational age. She was diagnosed with diabetes mellitus at the age of 6 weeks. She died at 4 months of age after rotavirus infection complicated by hepatorenal syndrome and multiorgan failure.

### Identification of the genetic alteration in the *EIF2AK3* gene

#### Genomic DNA sequencing

Based on the typical clinical symptoms and complications, genetic testing for WRS was initiated. Genomic DNA regions of the 17 exons and exon-intron boundaries of the *EIF2AK3* gene were amplified in 18 PCRs and bidirectional sequencing was performed. The PCR products of exon 11 showed characteristic patterns on the gel electropherogram: a healthy individual was represented by a 362-bp PCR product, in the case of *Patient 1* this was 352 bp, while in the case of the parents both bands were visible. DNA sequencing revealed a 12 bp deletion involving the exon 11 – intron 11–12 boundary (9 bp in the exon, 3 bp in the intron) combined with the insertion of a “T” and “G” nucleotide: g.53051_53062delinsTG (Ref Seq: NG_016424.1) (Fig. 1b). This alteration was present in homozygous form in the child and in heterozygous form in the parents (Fig. 1c and e).

Since the younger daughter of the couple died because of very similar symptoms, the presence of the disease was suspected in her case, too. Because of the early and unexpected death of this child only formalin fixed, paraffin-embedded tissue could serve as DNA source; therefore we isolated DNA from liver sections of *Patient 2*. Unfortunately, the PCR for exon 11 applied above did not work; therefore, a novel PCR method was developed for this region of the gene providing a much shorter PCR product (129 bp). Using this PCR, the genetic alteration observed in *Patient 1* and the parents could be identified also in *Patient 2*, in homozygous form (Fig. 1d).

Another 5 family members were tested for the presence of the described alteration. One of the younger twin brothers of the *Patients* and one daughter of the mother coming from her former relation was heterozygous for the genetic alteration, without any signs of WRS, while the others were negative (Fig. 1a).

#### mRNA/cDNA sequencing

**Fig. 3:**
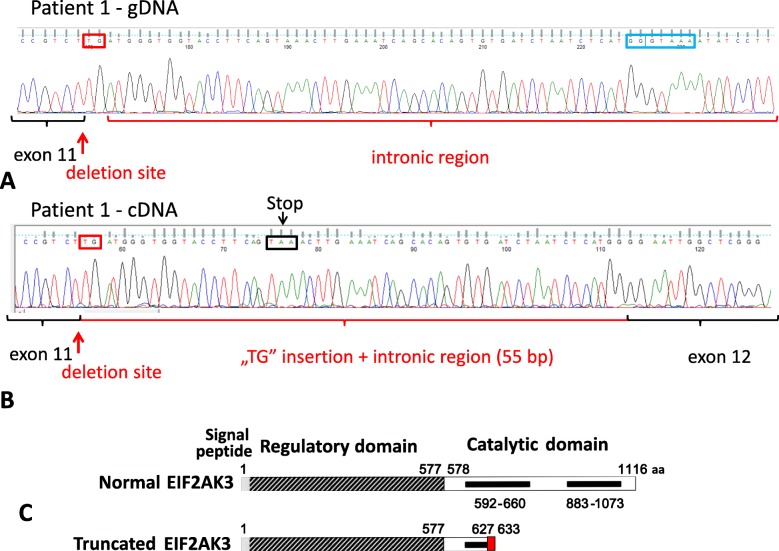
Consequence of the genetic alteration at the mRNA level determined by cDNA sequencing in the case of *Patient 1*. The cDNA of *Patient 1* was also sequenced showing that the genetic alteration caused the development of a novel splice site. On **a** the genomic DNA (“gDNA”) of *Patient 1* shows the location of the 12-bp-deletion, “TG” insertion (red box) and the place of the novel splice sequence (blue box) in the intronic part. On **b** the cDNA sequence of the patient presents the shortened exon 11, the intact exon 12, the inserted “TG” (red box) and the 55 bp intronic nucleotides, furthermore the stop codon (TAA, black box) resulted due to the frameshift in the reading frame. Translation of the altered mRNA might produce a truncated protein with 6 altered amino acids (position 627–632: DGWYLG) and a premature STOP codon at position 633 (**b** and **c**)

Since the identified deletion/insertion described at the DNA level involved the 3′ splice site of exon 11, its effect on transcription, protein sequence and expression was revealed by sequencing the cDNA of *Patient 1*. Special primer pair was designed involving sequences of exon 11 and exon 12, and the relevant fragment was amplified using the cDNA of *Patient 1* as template. Sequencing of this PCR product revealed that the observed deletion/insertion destroyed the former splice site and a new one was created (gg∣**gt**aaa) in the intron by 58 bp away from the original exon-intron border in the 3′ direction (Fig. 3a). The altered splicing resulted in a 55-bp insertion of the intronic sequence into the mRNA of EIF2AK3 (Fig. 3b). The result of this genetic alteration was a frameshift ending-up in a termination codon (TAA). The amino acid sequence was normal up to position 626 followed by 6 altered amino acids (DGWYLG) in the altered protein, and the position of the truncation was at amino acid 633 (p.Pro627AspfsTer7) (Fig. 3b and c).

### Consequences of the *EIF2AK3* gene variation at the protein level

#### Cloning and in vitro expression of the truncated EIF2AK3 protein

**Fig. 4:**
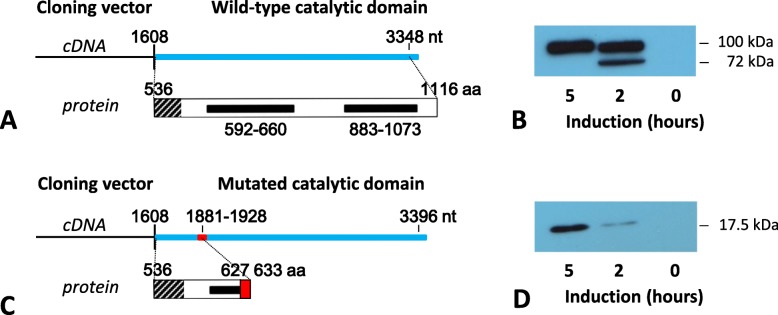
Cloning and in vitro expression of the truncated EIF2AK3 protein. **a** and **c** shows that the cDNA of the kinase domain of the wild-type and altered protein was cloned and expressed using the pTriex4Neo expression vector and the *E. coli* Rosetta2 pLysS bacteria. The transfected microbes were cultured and induced with 1 mM IPTG for 0, 2 and 5 h and after that were centrifuged and lysed. The wild type and truncated EIF2AK3 protein was detected by Western-blotting using His-tag specific antibodies, a peroxidase-labeled conjugate and a chemiluminescent substrate (**b** and **d**). The wild type protein was expressed in the bacteria partly as a 72 kDa and partly as a 100 kDa protein after 2 h of induction, while after 5 h only the 100 kDa form was visible. The larger form represents the autophosphorylated EIF2AK3 molecule (**b**). In contrast, − as it is presented on **d** - the cDNA reverse transcribed from the variant mRNA coded a truncated EIF2AK3 protein with a molecular weight of 17.5 kDa and no signs of phosphorylation could be observed

cDNAs of a healthy control and *Patient 1* spanning the cytoplasmic domain (amino acid 537–1116) of wild type and truncated EIF2AK3 were cloned and expressed in *E. coli* Rosetta2 pLysS. Cells from an uninduced culture after 5 h of culturing, and cells 2 and 5 h after induction with IPTG were centrifuged and lysed. The wild-type and truncated EIF2AK3 proteins were detected by Western-blotting. Two hours after the induction, the kinase domain of the wild-type protein was expressed as a 72 kDa and a 100 kDa protein, while after 5 h only the 100 kDa form was visible (Fig. 4b). The larger form represents the autophosphorylated EIF2AK3 protein referring to the wild type gene expression. In contrast, the cDNA reverse transcribed from the altered mRNA coded a truncated EIF2AK3 protein with a molecular weight of 17.5 kDa with no signs of phosphorylation, demonstrating that the genetic alteration resulted in a functionally inactive protein (Fig. 4d).

#### Expression of insulin and EIF2AK3 protein in the pancreatic tissue of Patient 2

**Fig. 5:**
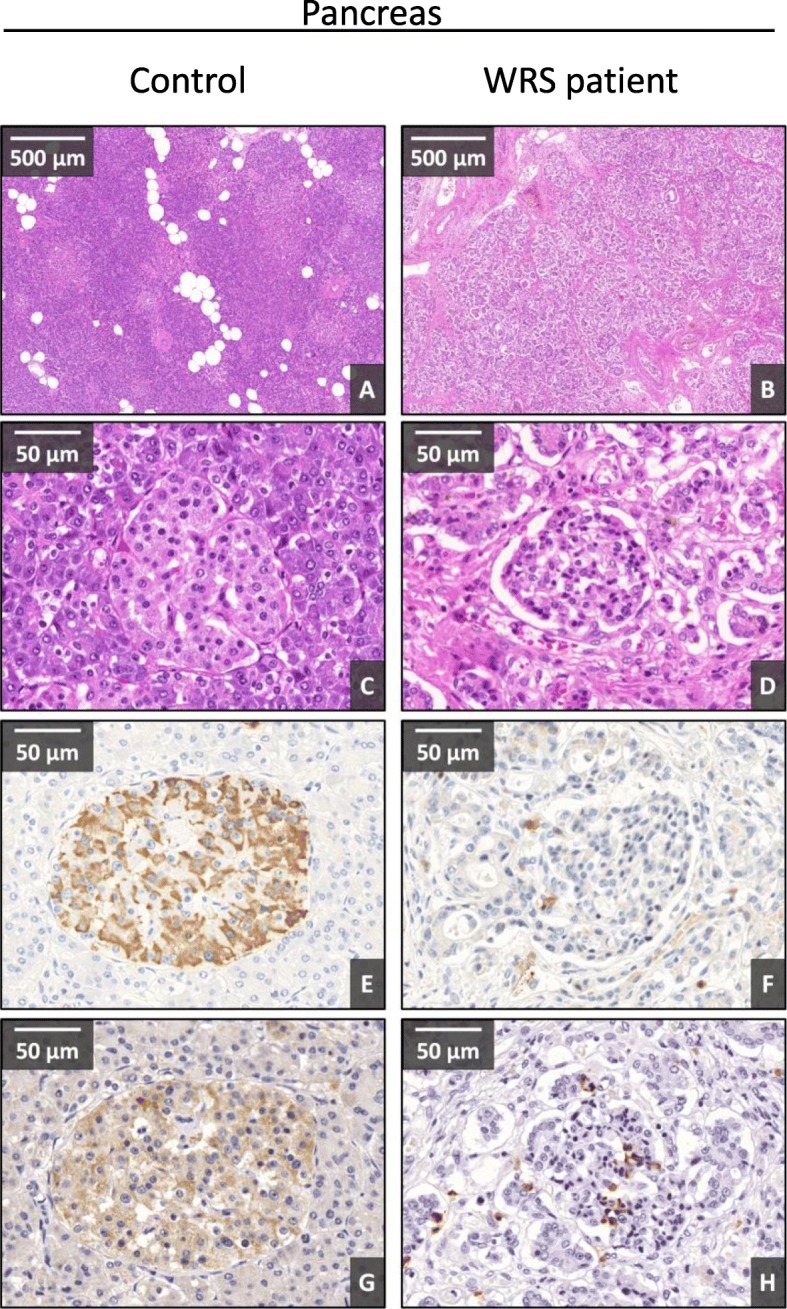
Expression of insulin and EIF2AK3 proteins in pancreas of the *Patient 2* with Wolcott-Rallison syndrome and a healthy control. 1st and 2nd row (**a**, **b**, **c**, **d**): H&E staining with different magnifications. 3rd row (**e**, **f**): Insulin immunohistological staining (chromophore: DAB - brown). 4th row (**g, h**): immunohistological staining by anti-EIF2AK3 antibody in Wolcott-Rallison syndrome and a control sample (chromophore: DAB - brown). The WRS samples present severe tissue injury with fibrotic remodeling with negative insulin staining in the Langerhans islands accompanied with negative EIF2AK3 protein expression. Control pancreas presents no tissue injury, and positive insulin-EIF2Ak3 immunoreactivity

In order to further characterize the expression of EIF2AK3 protein, immunohistochemical analysis of post mortem pancreatic tissue of *Patient 2, the younger sister,* was performed. The hematoxylin-eosin staining showed histological features of severe tissue damage with fibro-sclerotic scars. The parenchyma of the exocrine pancreas and the pancreatic ducts were atrophic, dissected by thick connective tissue branches in a spider web-like fashion compared to normal. The endocrine parenchyma was also atrophic; the diameter of Langerhans islands was small compared to control (Fig. 5a-d). The lack of β cells can be demonstrated by anti-insulin antibody based immunohistological staining. While the cells of the Langerhans islands in the control were positive for insulin with 50 to 80%, only less than 10% of the cells were stained for insulin in our child (Fig. 5e and f). The total absence of the intact EIF2AK3 protein was documented in the Langerhans islands of *Patient 2* upon comparison with a highly positive normal sample (Fig. 5g and h). We conclude that the alteration of EIF2AK3 protein in our WRS patient resulted in not only the defect of the PERK arm of ER stress and β cell injury, but also the severe damage and remodeling of the whole pancreas.

#### Altered expression of CHOP and GRP78 in the liver of *Patient 2* showed the presence of severe endoplasmic reticulum stress

**Fig. 6:**
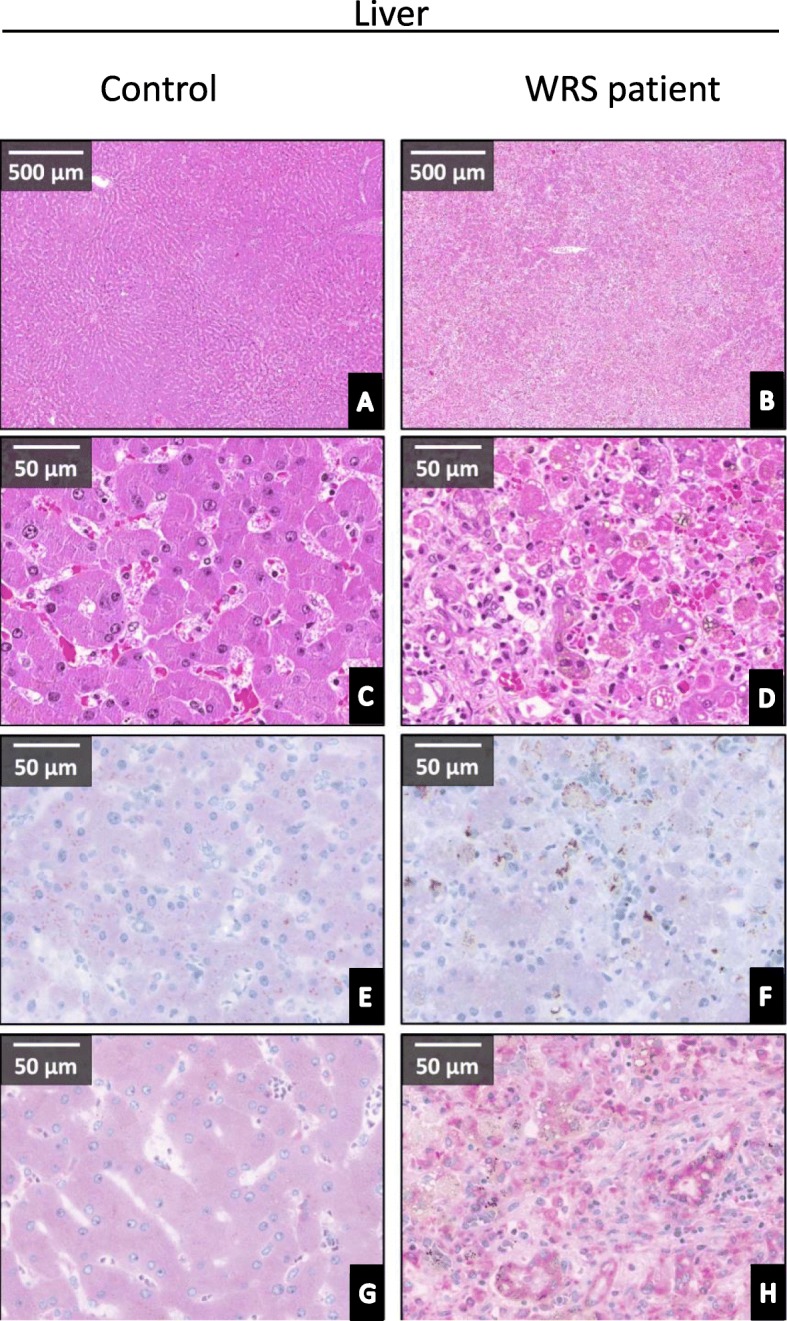
Expression of CHOP and GRP78 proteins in liver of the *Patient 2* with Wolcott-Rallison syndrome and a healthy control. 1st and 2nd row (**a**, **b**, **c**, **d**): H&E stain with different magnifications. 3rd row (**e**, **f**): CHOP immunohistological (IHC) staining (chromophore: VIP - purple). 4th row (**g**, **h**): GRP78 protein expression (IHC with VIP chromophore - purple). The tissue injury of the liver is severe, similarly to the pancreas in the WRS patient. The protective ER chaperon, GRP78 expressed tremendously accompanied with no CHOP expression in *Patient 2*. Control liver presents no tissue injury, extremely low GRP78 expression and a light immunostaining for CHOP

The hematoxylin-eosin staining also showed histological features of severe liver damage. The basic lobular structure was discohesive, most of the hepatocytes contained microvesicular lipid droplets and intracytoplasmic bile vesicles. Hepatocytes contained Councilman-body and Mallory-hyaline and their nuclear positivity disappeared at the edge of lobules, moreover the cytoplasm was homogenous and acidophilic. The portal region demonstrated sclerotic degeneration with massive lymphocyte accumulation (Fig. 6a-d).

Importantly, tremendous expression with intensive diffuse cytoplasmic positivity of the ER chaperon, GRP78 was observed in the hepatocytes of our WRS patient, while the control liver showed only light positivity (Fig. 6g and h). CHOP expression presented an opposite pattern. CHOP staining was negative in case of the patient, while light, granular positivity was detected in the hepatocytes of the control sample (Fig. 6e and f). We may summarize the pathological changes of the liver of our WRS patients as an example of the imbalance of cytoprotective chaperon and the damaging continuous ER stress.

## Discussion

Two children of a family with consanguineous parents, who carried a novel genetic alteration in the *EIF2AK3* gene (g.53051_53062delinsTG, Ref Seq: NG_016424.1; c.1878_1886 + 3delinsTG, Ref.Seq: NM_004836.7), showed the typical clinical scenario of Wolcott-Rallison syndrome, while one of their brothers, a half-sister and the parents of the patients were heterozygous carriers of the variant without any signs of the disorder. Both affected children had insulin-dependent neonatal diabetes mellitus with severe growth retardation, failure to thrive, anemia and neutropenia. In *Patient 1*, spondylo-epiphyseal dysplasia and slight hypothyroidism were also observed. Recurrent infections dominated the clinical picture in *Patient 1,* and in both children viral infection culminated in acute liver and kidney failure.

**Fig. 7:**
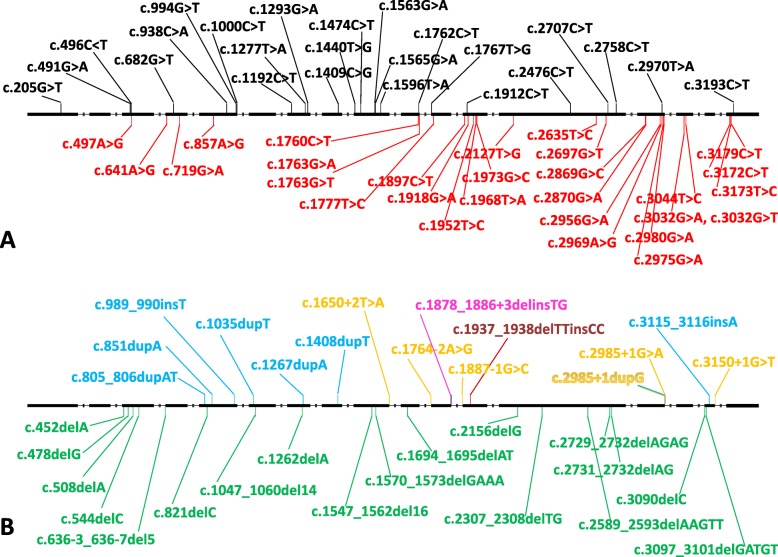
Location of the different genetic alterations in the *EIF2AK3* gene. Black boxes present the exons while dashed lines the introns of the gene. Vertical black lines on top of the gene are nonsense and red lines below the gene are missense variants (**a**). Green lines below the gene, blue and brown lines above the gene show the position of small deletions, insertions and indels, respectively. Yellow lines represent splice site alterations. The variant described in this study is presented in purple (**b**)

Based on the data of the Human Gene Mutation Database (HGMD), 88 different genetic alterations have been described in the *EIF2AK3* gene (Fig. 7) (HGMD® home page: www.hgmd.cf.ac.uk [20]) and majority of the cases (*n* = 52) represent single nucleotide substitutions (Fig. 7a), of which 24 result in STOP codon (nonsense variants) and 28 in amino acid change (missense variants). Beside the single nucleotide changes, 18 small deletions, 7 small insertions, 1 small indel (Fig. 7b), 1 complex rearrangement and 3 gross deletions are known to alter the sequence of the *EIF2AK3* gene. The distribution of the different genetic alterations along the *EIF2AK3* gene shows clear differences. Variants with pathologic phenotype, ending-up in an early STOP codon and a truncated protein, can be located in any exon, while missense variants are present dominantly in the kinase domain of the gene (exons 11 to 17) (Fig. 7a). Similarly, small insertions/deletions and splice site variants resulting in a frameshift and early termination of protein synthesis can be present in any exon (Fig. 7b). Regarding the genotype-phenotype association, there is hardly any correlation between the genotypes and the clinical scenario of WRS based on literature data. Even the same genetic alteration can be associated with different clinical signs and symptoms in separate individuals. Only the genetic variants located in the very end of the *EIF2AK*3 gene or single nucleotide substitutions (missense variants) in the 1st kinase domain can be associated with somewhat milder phenotype - longer overall survival or later onset of the disease -, most probably due to the slight remnant kinase activity of the altered/truncated protein [2, 3, 5, 9, 21].

In the background of WRS, 6 splice variants are described among the total of 88 genetic alterations. All of these WRS variants involve the most conserved nucleotides of the splice sites; the “GT” intronic sequence at the donor and the “AG” intronic base pair at the acceptor site: c.1650 + 2 T > A [3], c,1764 - 2A > G [22], c.1887 - 1G > C [23], c.2985 + 1G > A [17], c.3150 + 1G > T [24], and c.2985 + 1dupG [21]. The consequence of these splice site alterations represents exon skipping, less frequently the activation of a cryptic intra-exonic donor or acceptor splice site, or extremely rarely, the development of an intronic splice site resulting in aberrant inclusions of intronic parts as cryptic exons [25]. The translated protein may vary dramatically depending on the mature mRNA. The whole protein might be synthetized with some additional amino acids coded by the inserted intronic sequences or lacking some deleted exonic elements, or the whole protein might be intact excluding the skipped exon. If the alternative splicing creates a frameshift and/or an early STOP codon [25–28] there is a high chance for the development of a non-functional protein. The splice site alterations, the consequences of a DNA change, have to be evaluated at least at RNA level by RNA/cDNA sequencing or at protein level using different expression systems [25, 26, 28].

Intriguingly, the splice site variants described in the *EIF2AK3* gene have not been tested at RNA and protein level by far, most probably because they involved the most conserved nucleotides of splicing, and most probably these were the disease causing genetic alterations [3, 17, 21, 23, 24]. The novel variant described in our work is unique and has not been described previously in this gene. In vitro expression of the altered kinase domain’s cDNA revealed that the truncated protein was not autophosphorylated resulting in an inactive protein. Immunohistochemical analysis of the pancreatic tissue of *Patient 2* showed the lack of expression of the intact EIF2AK3 protein.

The major function of EIF2AK3 is to detect the accumulation of misfolded proteins in the ER, and - via phosphorylation of EIF2α - to reduce the rate of protein synthesis [15]. If the *EIF2AK3* gene is altered and the protein is not functional, the control of protein synthesis is lost, resulting in large amount of misfolded, aggregated proteins [16, 17]. Our results support this phenomenon, since the insulin staining in the pancreatic islet cells was diminished in the younger sibling together with the missing EIF2AK3 protein compared to a healthy sample where the normal expression of EIF2AK3 went along with normal insulin content in the β cells. This diminished insulin staining can be explained by the possible presence of insulin aggregates that might be less reactive to our anti-insulin antibody, or by the injury of insulin expressing beta cells due to their apoptosis induced by the overload of unfolded proteins.

The liver is the main target of ER stress. The activation of EIF2AK3 is an important pathway of ER stress. EIF2AK3 activation can be protective by reducing the misfolded protein load in the ER, but also can induce apoptosis by the CHOP dependent way [29]. Similarly to EIF2AK3, inositol-requiring enzyme 1 (IRE1) - the second arm of ER stress – can initiate the synthesis of protective chaperon proteins, but also may lead to apoptosis mediated by the apoptosis signal-regulating kinase 1/c-Jun N-terminal kinase (ASK1-JNK) pathway [30]. Finally, the third pathway, the activating transcription factor-6 (ATF6), stimulates the expression of protective chaperon proteins like GRP78 (glucose-regulated protein, 78 kDa) [31]. This was the case in our study. The insufficiency of the first arm of ER stress (the PERK pathway) resulted in the activation of the third one, ATF6 arm leading to GRP78 induction in the liver. Upregulation of GRP78 together with the absence of CHOP expression in the post-mortem liver sections of the WRS patient proved the diminished activity of the EIF2AK3 pathway. Signs of severe tissue damage and fibrotic reaction were visible on the HE staining, showing the presence of the continuous liver destruction.

Our data further support the importance of the genetic testing of the *EIF2AK3* gene in children with early onset diabetes who are born in consanguineous families. The early diagnosis of WRS can help the initiation of appropriate intervention therapy. The characteristics of our cases may allow us to hypothesize on a novel therapeutic approach; the introduction of ER stress inhibitors to delay combined en bloc liver-pancreas transplantation in WRS patients.

## Conclusions

The first WRS cases in Hungary are caused by a unique, novel indel variant involving the splice site of exon 11 – intron 11–12 boundary (g.53051_53062delinsTG, c.1878_1886 + 3delinsTG) resulting in the development of a new splice site. Due to the altered splicing, 55 nucleotides of intron 11–12 are inserted to the transcribed mRNA. The combination of these genetic events resulted in a frameshift and the development of an early termination codon at amino acid position 633 (p.Pro627AspfsTer7). The truncated protein is functionally inactive based on *in vitro* cloning and expression studies. In our WRS patient, intact EIF2AK3 was absent in the pancreatic islet *ex vivo*. The upregulation of GRP78 in the post-mortem liver sections of the WRS patient indicated an ongoing ER stress. The lack of the activity of the EIF2AK3 was associated with a unique liver histology characterized by diminished CHOP expression and severe liver injury.

## Data Availability

The datasets generated and analyzed during the current study are available in the GenBank repository (accession number to datasets: MT012628 and MT012629).

## Abbreviations

bp: base pair
CHOP: C/EBP homology protein
EIF2AK3: Eukaryotic Translation Initiation Factor 2-Alpha Kinase 3
ER: endoplasmic reticulum
gDNA: genomic DNA
GRP78: glucose-regulated protein, 78 kDa
IPTG: Isopropyl β-D-1-thiogalactopyranoside
PERK: PKR-like endoplasmic reticulum kinase
WRS: Wolcott-Rallison syndrome
